# From Affordances to Abstract Words: The Flexibility of Sensorimotor Grounding

**DOI:** 10.3390/brainsci11101304

**Published:** 2021-09-30

**Authors:** Claudia Mazzuca, Chiara Fini, Arthur Henri Michalland, Ilenia Falcinelli, Federico Da Rold, Luca Tummolini, Anna M. Borghi

**Affiliations:** 1 Body Action Language Lab (BALLAB), Sapienza University of Rome and ISTC-CNR, 00185 Rome, Italy; mazzuca.claudia@gmail.com (C.M.); chiarafini@gmail.com (C.F.); arthur-henri.michalland@univ-montp3.fr (A.H.M.); falcinelli.1615460@studenti.uniroma1.it (I.F.); darold.federico@gmail.com (F.D.R.); luca.tummolini@istc.cnr.it (L.T.); 2 IRCCS Fondazione Santa Lucia, 00179 Rome, Italy; 3 Department of Psychology, Université Paul Valéry Montpellier, EPSYLON EA 4556, 34199 Montpellier, France; 4 Institute of Cognitive Sciences and Technologies, National Research Council (CNR), 00185 Rome, Italy; 5 Department of Dynamic and Clinical Psychology, and Health Studies, Sapienza University of Rome, 00185 Rome, Italy

**Keywords:** body, action, abstract concepts, metacognition, sociality

## Abstract

The sensorimotor system plays a critical role in several cognitive processes. Here, we review recent studies documenting this interplay at different levels. First, we concentrate on studies that have shown how the sensorimotor system is flexibly involved in interactions with objects. We report evidence demonstrating how social context and situations influence affordance activation, and then focus on tactile and kinesthetic components in body–object interactions. Then, we turn to word use, and review studies that have shown that not only concrete words, but also abstract words are grounded in the sensorimotor system. We report evidence that abstract concepts activate the mouth effector more than concrete concepts, and discuss this effect in light of studies on adults, children, and infants. Finally, we pinpoint possible sensorimotor mechanisms at play in the acquisition and use of abstract concepts. Overall, we show that the involvement of the sensorimotor system is flexibly modulated by context, and that its role can be integrated and flanked by that of other systems such as the linguistic system. We suggest that to unravel the role of the sensorimotor system in cognition, future research should fully explore the complexity of this intricate, and sometimes slippery, relation.

## 1. Introduction

One of the aims of scientific explanation is to find unifying principles that hold true across different domains. When applied to the study of the human mind, one of these principles is the role of the sensorimotor system in different cognitive functions. Converging evidence has shown that different systems, traditionally related to the so-called “low-” and “high-level” cognitions, rely and build on the same system, i.e., the sensorimotor system. Theories of reuse [1,2] have clearly highlighted this aspect. Indeed, many authors have shown that language exploits and reuses structures that are characteristic of the most basic perceptions and action processes [3]. While this is, nowadays, widely shared across studies of various disciplines, the extent to which this relation is relevant or essential for semantic processing is still under debate.

Recently, it has been proposed that the activation of modality-specific systems and sensorimotor features in conceptual knowledge is not “an all-or-nothing affair, but rather a graded and flexible phenomenon that is sensitive to numerous factors, including task, context, and individual experiences” [4]. In the wake of this observation, the aim of the present contribution is to shed light on this complex relation. To this end, we focus on the “flexible” character of perceptual re-enactment, and we also attempt to refine the notion of “sensorimotor activation”, to account for language tout court.

We review research showing that sensorimotor grounding is a fundamental principle that concerns different types of processes. Specifically, we start by reporting studies and findings that address the relation between perception and action, with a focus on affordances. Next, we consider studies on conceptualization and language, with a focus on abstract concepts and words. In light of the evidence we present, we advance and defend two claims: (i) sensorimotor grounding can be flexibly adapted, depending on the context and (ii) sensorimotor grounding can differ in level and role, such that other experiential systems, such as the linguistic system, might complement the role of the sensorimotor system.

With the purpose of expounding on these aspects, we focus on studies that highlight two main characteristics of sensorimotor system involvement. First, we concentrate on the flexibility of the sensorimotor grounding during interactions with objects. We report evidence showing how the physical and social context and the current situation influence affordance activation (i.e., the motor recruitment during the observation of graspable objects). Then, we focus on the tactile and kinesthetic involvement in body–object interactions.

Next, we illustrate the flexibility of sensorimotor grounding in word use, with a special focus on abstract concepts, such as “freedom,” “thinking,” and “perhaps”, which are emblematic examples of how the sensorimotor, metacognitive, and linguistic systems might be integrated. Here, the term language is used to refer to semantics, but we move from the assumption that semantics and phonology might be strictly intertwined. In some cases, we refer more generally to linguistic experience, which also encompasses communicative and social aspects.

## 2. Flexibility of the Recruitment of the Sensorimotor System: The Case of Affordances, and Affordances and Language

### 2.1. Affordances among Perception, Action, and Social Practices

Affordance refers to the invitation to act that objects offer to us, for example, when we see a cup, we prepare to hold its handle [5]. The term has been widely re-evaluated in the last 20 years in the embodied and grounded cognition framework. Indeed, Gibson’s idea according to which affordances are neither subjective nor objective, entangling both perception and action, clearly fits with a paradigm that underlines the circular relationships between perception, action, and cognition. Although inspired by Gibson, seminal studies in cognitive psychology and neuroscience have diverged from his externalist perspective, stressing the role of the brain. In this perspective, affordances are forms of reactivation in the brain of visuomotor associations, for example, we typically experience cups with a handle and associate handles with a specific kind of grip. Along these lines, highly innovative studies have been performed, such as the early work by Ellis and Tucker (e.g., [6,7,8]. These studies focused on how we activate and potentiate these powerful associations during object observations regardless of tasks and contexts. More recently, the literature has been influenced by Cisek’s [9] competition model. In this view, action decisions are the product of competition in the brain of different affordances that are activated in parallel. For example, we might activate different affordances of an object depending on the context. A recent proposal by [10] broadens the notion of affordances, linking them to the skills of organisms; hence, for humans, affordances are linked to sociocultural practices. In this perspective, perception would consist of a sort of “openness to affordances” [10].

A variety of studies have recently shown that different tasks and contexts modulate the activation of object affordances. Studies have focused both on physical and social contexts, investigating their conjoint influence on affordance activation (for an overview, see [11], for a view integrating experimental and modeling evidence on affordances and mirror neuron system, see [12]). Some studies have investigated the neural underpinnings of the activation of affordances due to the physical context in which objects are located. For example, Wokke et al. [13] demonstrated, in an ingenious study with electrophysiological recordings, that object affordance activation was modulated by the context in which participants executed the task (e.g., a kitchen vs. a workshop). In addition, many cognitive and social neuroscience studies have investigated the role of social context during the observation of actions targeted at objects. For example, a seminal fMRI study demonstrated that context affects how we code high-level components of motor behavior [14]. Specifically, the same grasping movement performed in two different contexts (i.e., prompting an intention to drink or to clean) yielded a signal increase in the inferior frontal cortex as compared with when participants either observed the same movement detached from any context, or simply observed the context in isolation. The results pointed out how the mirror neuron system takes part in decoding actions’ intentions by simulating the motor planning more likely associated with a specific context. In another fMRI study, participants were asked to observe daily actions embedded in compatible, incompatible, and neutral contexts [15]. The results highlighted an increased activity in the inferior frontal cortex when the actions were framed in incompatible contexts. Interestingly, participants were also required to verbalize the observed actions, and instead of reporting the simple motor act, they described a sequence of motor actions embedded in a specific semantic context (e.g., “making pancake” instead of “cracking an egg”). Similarly, in a TMS study, ref [16] recorded MEPs from FDI and FCR muscles while participants watched videos about everyday actions with objects embedded in congruent, incongruent, or ambiguous contexts (e.g., pouring water from a full bottle into an empty glass or from an empty bottle into a glass full of water, and pouring water from a half-filled bottle into a half-filled glass). The videoclips were cut before the action ending, and participants were asked to predict the course of the observed action. The authors reported a modulation of FDI muscle, recruited during grasping action/observation, consisting of a selective decrease in corticospinal excitability during the observation of actions embedded in incongruent contexts as compared with congruent and ambiguous contexts. Together, these studies demonstrated that context helped to capture actions’ long-term goals, and played a crucial role in decoding action planning.

Consistently, behavioral studies have also investigated the influence of physical and social contexts on affordance activation. Examples of physical contexts are the presence of other objects, scenes, and situations in which objects are embedded, and the distance of objects from an agent’s body. Social context, instead, has been mainly studied manipulating the presence of another person [17,18] or more people, their collaborative vs. competitive attitude, the necessity or not to perform a joint action, and the social norms establishing the ownership of objects. Still, many questions remain unanswered. Among these, one question is whether we only activate affordances relevant for the current context and goal, or whether instead we always activate all affordances and only later operate a selection.

### 2.2. Affordances in Different Physical, Linguistic, and Social Contexts

Consistent with Cisek’s affordance competition hypothesis, but also with this broad view of affordances, we investigated the influence of both physical and social contexts on affordances. For instance, in our lab, we showed that the task influenced affordance activation, and that affordances emerged only when deep object processing was necessary. We found an affordance effect (compatibility between the handle position and the response key) when participants were required to process the shape of torches to determine whether they were upright or reversed. Instead, we did not find an affordance effect when they had to distinguish their color ([19], see also [20] for similar, independent results). We also showed that physical and social contexts influenced affordance activation. Using behavioral, EEG, and eye-tracking tasks, we found that the presence of other objects functionally related to the target and the hand of an agent potentially interacting with them facilitated affordance processing (e.g., [21,22,23]). In addition, we found that the part of the scene in which an object is situated evoked different grip-related affordances, for example, a corkscrew located in a drawer might evoke a power grip, whereas a corkscrew on a bottle might trigger a precision grip [24]. Affordances also vary depending on the goals of the participants, and can be modulated by age. In a recent study with novel and familiar objects, we found that young children responded to both novel and familiar objects in more creative ways than adolescents and adults, who responded to object affordances in more standardized ways (e.g., [25,26]). Finally, affordance activation is sensitive to our knowledge of ownership statuses (e.g., [27,28]). This knowledge is also grounded in the tendency to incorporate objects systematically associated with our body parts, such as rings, and consider them as part of our body [29]. An interesting case is represented by dangerous objects and their affordances. Their dangerousness is perceived differently and evokes different motor responses depending on their location in space and movement. To illustrate, Anelli et al. [30] presented neutral and dangerous objects dynamically, i.e., moving toward or away from the observer. They found slower responses when dangerous objects moved toward the participants, suggesting that they could evoke aversive affordances, leading to response inhibition. The importance of context in the processing of dangerous affordances appears even more prominent in a recent EEG study by [31], which demonstrated that dangerous affordances were not processed automatically, but were based on contextual information. For example, motor inhibition, with dangerous objects detected through a frontal N2 potential, was only present in a reachability task but not in a categorization task.

The converging evidence suggests that linguistic context also affects affordance processing. Possessing a label for a novel object can facilitate learning how to grasp it for use, but not how to move it. Semantic representations encode stable properties of tools, for example, sensorimotor properties such as shape and size, which remain rather invariant across contexts as compared with variable properties such as the current object orientation. Hence, language (semantics) contributes to ground conceptual information, such as proposed by the Label Feedback Hypothesis [32], and plays a direct role in motor learning [33]. For instance, it has been shown that reading verbs related to actions and functions induces a person to process affordances of objects close to their body faster than those distant from it, while reading verbs related to observing and pointing does not lead to any difference (e.g., [34,35]). In addition, action sentences (e.g., “grasp the brush”) elicit affordance related to the grip required by objects, while this is not the case for sentences related to observation [36]. Reading different verbs differentially activates affordances, and also listening to someone uttering action-related sentences can varyingly impact affordance activation. For instance, Gianelli, Scorolli, and Borghi [37] found that participants were faster in reaching close objects when a second agent said, “I take the object” as compared with “You take the object”. Therefore, the use of spoken language overall, and the linguistic choice (i.e., the use of the pronoun “I”), led to simulating a competition for the object’s possession. Consistently, grasping velocity was faster when the agent was an unknown person as compared with a friend. These results suggest an important role of both language and social context on affordance activation.

Currently, some recent studies are testing whether the broader context, such as the pandemic, can also impact affordance perception. Michalland et al. [38] are testing whether, in the current pandemic context, observing an object potentiates its affordances even if an unknown person has touched the object, thus, making it potentially contaminated. Similarly, Gianelli et al. [39] are addressing the possible effects of the pandemic on language and affordances. They are testing whether or not reading sentences describing actions involving objects that are potential carriers of the contagion (handle vs. toothbrush) evoke different affordances when the objects are embedded in public scenarios, and hence are potentially dangerous, such as the supermarket. Understanding whether social and cultural changes also impact established sensorimotor associations in such a short time may, therefore, provide us with further information about the extent to which affordances can be flexibly shaped.

Overall, these studies indicate that we recruit the sensorimotor system to plan and prepare our actions with objects, or even simply to simulate them. Remarkably, the involvement of the sensorimotor system occurs in a highly flexible and context-dependent way. We believe that one of the challenges in the next few years is to determine, with precision, the elements at play in the competition for the emergence of affordances [11]. This would allow us to predict when and in which circumstances different kinds of affordances emerge. Our life takes place in a kaleidoscopic influence of many factors. Provided that the sensorimotor system is always involved, at which level is it involved? When do more stable affordances prevail, and when, instead, do the cues derived from the context prevail? In the following sections, we focus on sensorimotor activation patterns of a specific modality, i.e., the sense of touch, to provide examples of its flexible and context-bound involvement.

### 2.3. Affordances and the Differential Involvement of Tactile and Kinesthetic Modalities

During a potential or real object manipulation, sequences of actions with different goals are planned, simulated, and performed. Action phases are divided into events in which we anticipate and experience different types of contact with the objects. From such sensorimotor events, somesthetic signals are generated through actual stimuli or sensorimotor simulations. These somesthetic signals can involve interoceptive, tactile, or proprioceptive signals (e.g., [40,41]). Among these, the involvement of tactile and proprioceptive modalities to detect various properties of objects such as softness, roughness, temperature, or curvature, and of events such as grasping, lifting off a surface, or slipping through fingers are of primary importance and are now well documented (e.g, [42,43,44,45,46,47]). Those tactile and proprioceptive signals are involved in many human activities [48] and support multiple cognitive processes entailing body–environment interactions.

Indeed, tactile and kinesthetic consequences are integrated with action codes to select the effector and the force needed to perform an action (e.g., [49,50,51]), movement trajectories [52,53,54], and to refine the generative model used in active inferences [55]. Therefore, potential nociceptive tactile consequences derived from object dangerousness (e.g., [30,31]) in addition to the state of the body can be considered in the selection of action codes. For instance, presenting a picture of an injured hand as compared with that of a healthy hand leads participants to produce slower responses, especially when they have to grasp a response device [56]. However, the complexity and variability of tactile and kinesthetic signals (e.g., [57,58]) may also lead the cognitive system to introduce some flexibility into the integration of such signals. For example, Michalland et al. [59] showed that the impact of object dangerousness, object position, and body state depended on the hand used to produce a response; dominant (right) hand responses took into consideration all these features, while left hand responses did not take into consideration the body state.

A focus on the tactile and kinesthetic modalities can, thus, deepen our understanding of affordance flexibility and the features exploited in this body-environment bridging. For example, while the shape and spatial attributes of objects are features that may induce affordance activation (e.g., [7,19]), we know little about the impact of other object features. With the purpose of deepening this aspect, Michalland et al. [60] investigated the force exerted when facing pictures of objects varying in weight or softness. They showed that participants modulated the exerted force when using their nondominant (left) hand, but not with their right hand. Thus, the features of objects taken into consideration to perform an action may vary depending on the hand used (see also [61]). In that sense, the flexibility of affordance activation can also be related to the flexibility of the environment features selected to produce an action. This flexibility would, thus, bias the weight of the various sensorimotor modalities in the competition model proposed by Cisek [9].

The differential involvements of tactile and kinesthetic features in physical body–object interactions underscore the flexibility of sensorimotor grounding of cognitive processes. Just as the various dimensions of tactile and kinesthetic modalities can be varyingly involved in body–object interactions, different bodily and sensorimotor features can be involved in language processing, as a particular word or concept recruits sensorimotor grounding sources through its referent and/or through the actions of talking and listening (e.g., [62,63,64]). In the following section, we describe how these bodily components are responsively co-opted for object concepts and also for more complex and abstract concepts.

## 3. Different Levels of Involvement of the Sensorimotor System, and Integration with the Linguistic System: The Case of Abstract Concepts

Over the years, neuropsychological findings have shown that some patients can successfully recognize some categories of things but not others, fueled a longstanding debate on the organizing principles of conceptual organization and its neural underpinnings. For instance, in a seminal study, Warrington and Shallice [65] reported the case of JBR, a patient with temporal lobe damage that provided correct descriptions for nonliving artifacts while exhibiting a poor knowledge of living things. This type of evidence (see [66]) initially gave rise to two main counterposed classes of theories. On the one hand, models related to the Sensory-Functional Hypothesis (e.g., [65,67,68,69] have proposed that the ability to identify living and nonliving things depends upon two separate systems, i.e., a visual semantic system and a functional-associative system. In this account, living things and natural kinds are mainly processed and recognized by means of their sensory properties (e.g., shape, color, and texture), while nonliving things and artifacts are mostly represented relying on functional properties (e.g., prototypical use and functions entailed). On the other hand, models that refer to the Distributed Domain-Specific Hypothesis (DDSH, (e.g, [70,71,72,73,74]) have proposed that the organizing principle of knowledge is determined by the role that categories have played in evolutionary history. Properties of these categories are stored together in the brain, and therefore category-specific deficits should result in uniform impairments for visual and functional attributes of a concept. In addition, the categories of category-specific deficits are fractioned, for instance, the categories of animals (animate biological objects) and those of fruits and vegetables (inanimate biological objects) can be affected by brain damages independently of each other [75].

Nowadays, there is consistent evidence that the meanings of some concrete words depend on modality-specific brain regions, encoding specific perceptual components of the terms such as, among others, color [76], taste [77], and smell [78]. Recent scientific endeavors have also provided a sort of brain topography of concepts, based on weightings of different types of perceptual and experiential properties (see e.g., [79,80]). However, research strands that are skeptical of a “strong” version of embodiment (e.g., [81]) have suggested that the activation of sensory-motor areas in semantic tasks might just be a byproduct of lexical processing, instead of constituting its foundation. In this perspective, meaning is represented at an abstract, symbolic level that interacts with sensorimotor information when conceptual instantiations are elaborated. This view is bolstered by neuropsychological findings on apraxia patients, whose semantic knowledge on how to use objects is intact, while at the same time showing an impairment in the practical use of objects. Therefore, while the engagement of modality-specific systems in semantic processes is widely documented, there is still controversy in the field concerning its relevance.

### 3.1. Grounding of Abstract Concepts in the Sensorimotor System

While the role of sensorimotor aspects involved in the processing of objects, actions, and object concepts is now well established, only recently has the scientific community started to acknowledge the importance of sensorimotor components in the representation of more abstract entities. Traditionally, concepts have been divided into two general classes, i.e., concrete and abstract concepts. Concrete concepts refer to physical and perceivable entities in the world (e.g., hammer). Converging evidence has shown that concrete concepts are acquired earlier in life [82], and processed and remembered faster [83]. Conversely, abstract concepts (e.g., justice), i.e., concepts referring to ideas or entities which are not experienced through the five senses, have a general disadvantage in response times and are acquired later in life (see [84]). Research focusing on concrete concepts has widely documented the entanglement between conceptual processing and systems devoted to perception and action (see e.g., [66,85]), as well as the role of sensorimotor features in language comprehension (e.g., [86,87,88,89,90]). In addition, concrete concepts are said to be acquired primarily through physical interaction with their referents, while abstract concepts are generally acquired and represented via linguistic associations (e.g., explanations or examples (e.g., [91,92,93]). While most studies have focused on children and adults, recent results on word comprehension in infants have confirmed that abstract words are learned later and often in conjunction with the acquisition of critical social abilities, such as joint action [94]. Furthermore, studies on word production of infants suggest that possessing abstract words in early vocabulary can enhance later language acquisition (e.g., [95,96]). The importance of linguistic associations for abstract concepts is also testified by research on the elderly, especially by evidence showing that, likely because they rely on language, abstract concepts deteriorate less than concrete concepts with age (for a review, [97]).

#### 3.1.1. Different Abstract Concepts Are Couched in Different Modalities

Although most of the evidence in favor of a causal role of sensorimotor simulations in conceptual processing has come from studies dealing with concrete concepts, nowadays, there is a growing interest in assessing sensorimotor components engaged in abstract knowledge as well. Mathematical knowledge, for example, has been the remit of several studies that have documented the activation of specialized neural correlates (especially for concepts denoting numerosity, see (e.g., [98,99]). Along these lines, proponents of embodied cognition posit that numerical and mathematical knowledge is grounded in motor processes related to the habit of finger counting and spatial associations (e.g., [100,101,102]). Consistently, some studies have shown that tiny and large numbers evoke different gestures (e.g., [103,104]). We documented these associations in various behavioral and kinematics studies, where we found that participants computed more additions than subtractions when performing an ascensional movement, moving rightward, and moving in a circular clockwise way (e.g., [105,106,107]). For example, Anelli et al. [107] asked participants to subtract or add three to a starting number for 22 s while either turning leftward or rightward from a straight walking path, and then to report the result aloud. They found that participants provided more correct answers in “congruent” conditions (i.e., subtractions leftward or additions rightward). These results are in line with the idea that small numbers are embodied and spatially related [108].

We also found that object affordances and task-irrelevant hand actions enhanced the sensitivity to numerical magnitude, and that numerical magnitude modulated grasping (e.g., [109,110]). These results document the strict relationship between the processing of abstract concepts (numbers) and their sensorimotor basis. While there is plenty of evidence of the sensorimotor grounding of numbers, it is possible that, especially for large numbers, not only sensorimotor and spatial components but also linguistic aspects (semantics) play an important role in their representation.

Emotional concepts have also been found to activate a widespread network of brain areas, mostly related to emotion processing (e.g., fronto-parietal regions) together with motor and premotor areas (see [111]). For instance, Moseley and collaborators [112] found, in an fMRI study, that even abstract emotional words with low scores on ratings of sensorimotor activation (e.g., hate and gibe) activated the precentral cortex, overlapping with areas activated by arm- and face-related verbs. More recently, in a meta-analysis comparing patterns of neural activation for different kinds of abstract concepts, Desai, Reilly, and van Dam [113] found that the representation of emotional, numerical, and moral concepts and concepts referring to Theory of Mind (TOM) was spanned over different brain regions. Nonetheless, each subcategory was associated with uniquely identifying areas. The specificity of modality-specific brain regions co-opted in the elaboration of abstract concepts was also illustrated in an fMRI study that compared patterns of activation of 64 abstract concepts, distinguished according to their specific features (motor, e.g., fitness vs. visual, e.g., beauty, [114]) in a lexical decision task. The results showed that processing motor abstract concepts activated areas usually found active in the execution of hand movements (left precentral and postcentral gyrus), whereas visual abstract concepts triggered the activation of lingual and fusiform gyrus that are often reported during the observation of object scenes. Finally, despite the fact that the abstract domain of object ownership has been proposed as the hallmark of disembodiment [115], recent studies have shown that explicit knowledge of the ownership status of objects interacted with multisensory and motor processes in surprisingly direct ways. As suggested above, it has been shown that knowing whether a graspable object (e.g., a cup) is “mine” or not differentially modulates the automatic potentiation of actions towards it (affordance activation [27]). In a simple grasp-to-lift task, such knowledge can alter the kinematic profile of movements in ways that suggest an automatic resistance to interact with objects owned by others [27]. Knowledge of the ownership status of objects can also influence the linguistic choice of spatial demonstratives such as ‘‘this’’ and ‘‘that’’ in subtle and unconscious ways [116]; participants tend to use ‘‘this’’ more often to refer to objects that they own than to objects owned by someone else. Intriguingly, a recent study has also provided initial evidence that the ownership status of an object can also affect the multisensory representation of the space around the body (the peripersonal space [117]), as measured by the enhancement of visuotactile interaction effects when manipulating objects that belong to the participant but not with objects belonging to someone else. Finally, intriguing evidence from a somatoparaphrenic patient denying ownership of her left hand revealed that she also displayed selective disownership of objects typically associated with it (e.g., a wedding ring, a garnet ring, a watch, etc. e.g., [29,118]). Taken together, these studies strongly suggest that the abstract conceptual domain of ownership may in fact be, at least partially, grounded and profoundly shaped by our sensorimotor experiences [84]. These findings support the idea that the representation of abstract concepts—similarly to that of concrete concepts—also recruits sensorimotor neural areas, while at the same time pointing to the composite and heterogeneous character of the category of abstract concepts.

Behavioral and linguistic results also advocate for a more fine-grained perspective on abstract concepts. Methods typically used to identify underlying features of conceptual representation (e.g., feature listing, ratings, and typicality judgments) have highlighted how different aspects (e.g., internal, perceptual, and social) concur in the representation of abstract concepts, sometimes overlapping with more concrete features. To illustrate, Connell and Lynott [119] found that, across more than 500 English words, ratings of concreteness and perceptual strength (i.e., the extent to which a concept is experienced through one of the five senses) did not always align. Specifically, concepts related to taste (e.g., bitter) or sound (e.g., noisy) experiences were found to have strong perceptual components while being highly abstract. Importantly, the authors also showed that perceptual strength scores outperformed traditional psycholinguistic measures such as concreteness and imageability in lexical decision and word naming tasks. Along the same lines, Troche and colleagues (e.g., [120,121]), in two large rating studies, reported that abstract concepts were characterized mainly by affective, social, and moral aspects. However, concepts with higher affective emotional components (e.g., chocolate and trust) tended to cluster together irrespective of their abstractness level, further suggesting that the distinction into abstract and concrete classes of concepts alone might not suffice to capture all grounding sources efficiently. In addition, a recent study by [122] shed further light on other modality-specific grounding mechanisms for abstract concepts, showing a predominant role of interoception in abstract conceptual representation and processing. Internal grounding was also found to be one of the latent factors explaining abstract concepts’ representations in a large rating on Italian abstract words [123]; interestingly, the ”inner grounding social” factor included the relation of abstract meanings with the mouth effector, together with emotions, metacognition, and interoception (see also [124]). Crucially, the role of sensorimotor grounding also varies within abstract concepts. Villani et al. [123] showed that the latent sensorimotor factor characterized primarily physical, spatio-temporal, and quantitative (PSTQ) abstract concepts (e.g., reflex). The inner grounding factor played a more critical role for self-sociality (SS) (e.g., politeness), and emotive/inner states concepts (EM) (e.g., anger). Philosophical-spiritual (PS) concepts (e.g., value) qualified as more abstract than the other concepts. In addition, recent studies have revealed that the expertise of participants and the culture might influence the perceived role of sensorimotor features. In a rating study, law experts judged institutional concepts as involving more the emotional dimension and the sense of touch than a control group; since touch is typically associated with concrete concepts, these results suggest that expertise might contribute to rendering abstract concepts more concrete [125].

Feature listing tasks also offered hints into perceptual and sensorimotor components of abstract concepts (for a review, see [126]). For instance, Harpaintner, Trumpp, and Kiefer [127] asked participants to generate features for 296 abstract concepts, and found that, while internal, emotional, and social aspects were especially relevant, sensorimotor features were also present. Aside from the results obtained with more classic ratings and feature production tasks, recent studies using novel, interactive methods have reached similar conclusions. Villani, Orsoni et al. [128] asked participants to respond to a sentence containing an abstract or concrete concept as if they were engaging in a conversation. As compared with concrete sentences, abstract sentences evoked primarily inner properties, but they also yielded sensorimotor ones; specifically, physical, spatio-temporal, and quantitative (PSTQ) abstract concepts yielded more sensorimotor features than the other abstract concepts. Notably, abstract sentences also led to more interactive exchanges, characterized by more questions to the fictitious interlocutor [128]. Further studies carried out in our lab have confirmed this interactive component of abstract concepts. For instance, in one study currently in preparation, we asked participants to create a post for Facebook and Twitter, starting from different types of concrete and abstract concepts [129], while in a different study we investigated mind wandering in children and adolescents who received concrete and abstract words as cues [130]. The participants were submitted to a boring task, in which they observed figures and pressed a button when they detected a circle (10% of the times); near the figures, concrete and abstract words were displayed. In some trials, participants were suddenly invited to report their thoughts, indicating whether they referred to the task or not, evaluating their vividness, indicating whether they referred to the past, present, or future, etc. The preliminary results from these studies have indicated that more abstract concepts evoked more questions and interactive exchanges than concrete concepts.

#### 3.1.2. Culture and Language Shape Bodily Components of Abstract Concepts

Cross-cultural studies notably illustrate the variable integration between sensorimotor components and abstract concepts. Indeed, cultural backgrounds represent the natural scaffolding where the relation between language and body is flexibly shaped [131]. Evidence from ours and associated labs also confirms that sensorimotor and perceptual components are flexibly incorporated into abstract concepts, depending on specific experiences or cultural settings. In a recent study, Italian and Persian participants were asked to read concrete and abstract sentences, i.e., sentences referring either literally (i.e., concrete sentences such as “she hits the child”) or metaphorically (i.e., abstract sentences such as “she grasps a concept”) to motor actions, preceded by a video displaying a movement that could be congruent or incongruent with the action described in the sentence. Participants were asked to re-execute the movement observed, and then to evaluate whether the sentence made sense or not. We found that, in the Italian sample, response times were faster with concrete sentences than to abstract sentences, especially in the congruent condition. In contrast, Persian participants responded faster to abstract sentences than to concrete sentences in the congruent condition, while concrete and abstract sentences did not differ in the incongruent condition. The results confirm that language and action are strongly integrated, but that this integration might be modulated by culture. In fact, the facilitation we obtained with concrete sentences in the Italian group was likely due to the higher integration of language and gestures in the Italian culture [132]. The results also suggest that culturally acquired habits might strongly influence concrete and abstract language grounding in the sensorimotor system. In parallel, studies on sign languages have confirmed that different relationships between abstract concepts and body parts might be salient depending on the culture and signed language [133]. Similarly, a concept such as gender, which cannot be considered strictly abstract, or concrete, displays differing characterizing features as a function of participants’ previous experiences and cultures. To illustrate, Mazzuca et al. [134] asked a sample of Italian “normative” (i.e., monosexual and cisgender) and “non-normative” (i.e., plurisexual and gender diverse) participants to provide free associations to the word genere (“gender”). We found that, while “normative” participants mainly stressed aspects related to a binary, more concrete conception of gender (e.g., woman and man; female and male), “non-normative” participants mostly produced sociocultural, more abstract features (e.g., construction, queer, and fluidity). Preliminary results by [134] also indicated that more abstract or more concrete features of gender might be differentially relevant depending on cultural and social aspects. For instance, when asked to list words referring to gender, Dutch participants more frequently mentioned words linked to bodily and perceptual components of the concept, for example, breasts, vagina, penis, and hormones. Conversely, Italian participants focused more on aspects mediated by sociality and culture, mentioning words such as discrimination, construct, and patriarchy more frequently. The results from the following rating study, in which participants were asked to rate a set of abstract and concrete words in terms of how much they were related to gender, supported the idea of different levels of abstractness in the conceptualization of gender between Dutch and Italian speakers. In fact, Dutch participants rated more concrete words as more related to gender, whereas Italian participants showed the opposite pattern. Finally, recent studies have indicated that, likely because of their more substantial reliance on the linguistic than on the sensorimotor system, abstract concepts vary across languages more than concrete concepts (for a review, see [135]). Along these lines, recently, we asked Italian, Iranian, and Israeli participants to sort concrete and abstract nouns into groups and to freely label each group they created [136]. The results revealed a higher variability of abstract concepts as compared with concrete concepts, both within individuals of the same culture and across cultures and languages.

To summarize, abstract concepts seem to be primarily characterized by dimensions such as affect, internal states, and social components; perceptual and sensorimotor features are also implicated in their grounding. Significantly, however, the role of internal and external grounding is flexibly modulated by the context, i.e., the language, culture, and current situation, such as a recent study indicated on the impact of the COVID-19 pandemic on conceptual organization [137].

### 3.2. Grounding of Abstract Concepts in Metacognition

In addition to internal domains such as affect and interoception, abstract concepts might also be grounded on metacognitive processes in which higher-level systems monitor and control other object-level mental states and processes such as perception, memory, learning, and reasoning [92]. Although often mentioned, so far, the role of metacognition in grounding abstract concepts has not been systematically explored, and discussions on its involvement have typically been limited to conceptual domains with explicit meta-level content such as mental state concepts; moreover, its integration with the sensorimotor systems has been mostly neglected. However, consider again how a basic understanding of “mine”, “yours”, and other concepts of property ownership might develop. We have already illustrated recent evidence of a direct grounding of this abstract knowledge domain on the sensorimotor system. Despite this connection, it has been argued that the semantic core of ownership is ultimately related to the unobservable, and thereby abstract notion of “control” [28]. Tracing a plausible cognitive development of this control-based view, it has been hypothesized that concepts of possession and ownership develop as a byproduct of the intrinsic motivation of children to effectively interact with the environment, and the need of infants, during their first two years of life, to identify the objects in their environment that give rise to feelings of efficacy and personal control to keep them apart from those that instead thwart such feelings [138]. From the child’s perspective, the former class of controllable objects becomes the category of objects that are understood as “mine”, while the latter class includes those that are not understood to be theirs. Casting this proposal in contemporary computational frameworks of reinforcement learning might reveal that such a curiosity-based exploration of new skills relies on monitoring one’s competence improvement (or lack thereof), which is a fundamental metacognitive learning signal (e.g., [139,140]). Thus, in principle, even metacognitive processes that monitor and control lower levels of sensorimotor processes can provide the kind of information that can be used to develop and ground higher-level abstract concepts.

### 3.3. Abstract Concepts, Language, and Their Relation with Mouth Motor Areas

In addition to interactive experiences, abstract concepts tend to be acquired mainly through linguistic inputs [141]. In fact, while concrete concepts generally refer to things that can be experienced through the senses, and therefore indicated and manipulated, this is not the case for words denoting abstract entities. This is tightly related to a cardinal aspect of abstract concepts, i.e., the fact that they collect under single linguistic label entities and situations that may have very few common features (see the notion of low dimensionality in Lupyan and Mirman [142]). This characterizing feature of abstract concepts makes them more difficult to acquire through sensorimotor and perceptual interaction as compared with concrete concepts. Indeed, when explaining to a child what a “chair” is, it would be sufficient to show them the object. In this case, despite the fact that chairs can be of different kinds, colors, and materials, they typically share common features that allow a child to abstract away from specific instantiations of a chair to form a more general category. Conversely, when explaining what “justice” is, we would need to resort to linguistic explanations such as “fairness in the way people are treated” [143]. This definition applies to a variety of situations that can potentially be indicated to a child as instantiations of “justice”, often very different among each other. Therefore, linguistic labels and explanations provide a sort of “conceptual glue” for abstract meanings. Studies investigating the modality of acquisition of abstract and concrete concepts in children have confirmed this intuition. Abstract concepts have, in fact, been found to be primarily acquired through linguistic interaction (might that be spoken or written language), whereas concrete concepts have been found to be acquired mainly via perceptual processes [141].

In keeping with that, according to multiple representation proposals, the representation of abstract concepts should massively rely on the linguistic system [144,145,146,147]. The specific recruitment of linguistic information in the representation of abstract concepts has been confirmed by rating studies showing that abstract concepts are judged to be more associated with the mouth effector as compared with concrete concepts, which in turn are more associated with hands or other effectors eliciting action patterns ([124], see also [123]). Ratings and behavioral studies have further suggested that this association with the mouth is particularly marked with some kinds of abstract concepts, such as mental states and institutional concepts (e.g., [124,125]).

Behavioral studies in which participants were asked to use the hand or the mouth to deliver responses have established the connection between mouth activation and abstract concepts (for reviews (e.g., [144,147]). Borghi et al. [148] and Granito et al. [149], both implemented novel paradigms to investigate how we form new conceptual categories from elements, i.e., geometric shapes or names, never experienced before. In the study by Borghi et al. [148], after having manipulated or interacted with new objects, and then formed conceptual categories, participants were submitted to a property verification task in which they were required to indicate whether a feature belonged to a specific learned concept. Participants were faster with abstract concepts when using a microphone to respond and with concrete concepts when pressing a button on the keyboard. Similarly, in the study by Granito et al. [149], participants were submitted to a categorical recognition task after learning verbal categories from new objects and names. The results indicated that responses were faster when the words were abstract and the answer was delivered with a microphone, for participants who have benefited from linguistic training. The advantage of using the microphone over the hand when processing abstract concepts was found, for the first time with real words and sentences, by Borghi and Zarcone [150], in a study where participants had to decide whether a concrete or abstract word matched with a definition. Finally, Mazzuca et al. [151] confirmed the same effect in a word recognition task but not in a lexical decision task, using a slightly different paradigm. Specifically, they designed two experiments in which participants responded to abstract, concrete, and emotional words either by pressing a button with the hand or with the mouth (Experiment 1), or by pressing a pedal while responding to catch trials with a hand-mouth button (Experiment 2). In both experiments, abstract words were responded to faster when the mouth effector was engaged as compared with when the hand effector was engaged. However, this only held for the word recognition task, while in the lexical decision task of both experiments, there was no difference between the mouth and hand conditions. The latter probably failed to replicate the results because the task might have been too shallow, suggesting that the activation of mouth motor areas might boost the semantic processing of abstract words at a deeper level. Other studies have outlined interference effects, which emerged when the mouth was occupied while performing a task, as in the study by Villani et al. [152]. Here, participants were asked to chew gum while evaluating the difficulty of words, and such manipulation resulted in an increase in the perceived difficulty of concrete but not abstract concepts.

Neural evidence from TMS and fMRI studies has further elucidated the role of mouth motor areas in processing abstract meanings. In a TMS study, Scorolli et al. [153] had participants process sentences composed by abstract and concrete nouns and verbs. The early activation of hand-related areas with concrete concepts and delayed activation of the same areas with abstract verbs was likely due to a cascade effect of early activation of the topologically contiguous mouth motor areas. Regarding the fMRI studies, Sakreida et al. [154] compared concrete and abstract expressions and found that abstract sentences consistently activated the anterior part of the left middle temporal gyrus, one of the language system nodes. While specific patterns of activation of mouth-related areas have previously been reported for emotional words (e.g., [155,156]), Dreyer and Pulvermüller [111] extended previous findings to mental abstract words (e.g., logic). Scanning hemodynamic activity within the motor system during a passive reading task, they found a stronger activation of face motor areas for mental abstract words as compared with emotional abstract words, which, instead, activated different foci of motor areas (e.g., hand, leg, and mouth) to the same extent. Together, such evidence corroborates the hypothesis that acquiring and processing abstract concepts might request a more substantial linguistic contribution as compared with concrete concepts, expressed by the facilitating or interfering effect of mouth motor area activations observed in various experimental conditions.

One question that might arise concerns the centrality of mouth involvement in linguistic processing and, more crucially, in the processing of abstract concepts. To address this issue, it might be worth considering the debate on articulation in inner speech, in which there are two opposing viewpoints. According to one viewpoint, motor articulation is necessary for inner speech to occur; according to other views, mental simulation of speech would involve only the first stages of speech production, before speech articulation – hence, inner speech would be abstract and not necessarily articulated (review in [157]). As proposed by some authors (e.g., [158]), speakers can monitor the degree of articulation of inner speech, which can vary dynamically. Therefore, depending on the task and context, inner speech can be either articulated or not [159]. These insights might be of assistance for elucidating the role of the mouth in the processing of abstract concepts. The activation of mouth motor areas has been documented in many studies that have investigated abstract concepts, which certainly indicates the relevance of linguistic experience for abstract concept processing, which might be linked to the use of inner speech. However, language experience might be evoked even in the absence of mouth involvement, as suggested by research on inner speech articulation. Therefore, although our results indicate that responding with the mouth facilitates the elaboration of abstract words, and that occupying the mouth might hinder abstract concepts’ representation (see below), based on the evidence we have collected so far, we cannot conclude that mouth involvement is necessary and constitutive for abstract concept processing, granting the comprehension of abstract word meanings. Further research should investigate this.

While the studies discussed so far attest the entanglement between linguistic motoric components and abstract concepts in the context of online language processing or in task mirroring processes of conceptual acquisition with novel stimuli, they do not directly target the acquisition of real abstract words. One way to address the role of linguistic motoric components in abstract conceptual knowledge emergence is to look at the developmental pathway of this connection. In the following section, we report studies that we conducted that might offer key insights into this undertaking.

#### Mouth Engagement and Abstract Concepts in a Developmental Perspective

Behavioral data collected with adults responding to abstract stimuli indicate that the mouth motor system is consistently involved in the processing of abstract concepts. In addition, as already mentioned, abstract concepts are mainly acquired through linguistic inputs and social interactions (e.g., [141,144]). Given the significance of linguistic simulations occurring in the mouth motor area for abstract concept acquisition and processing, one might wonder whether consistently inhibiting such processes could lead to a selective impairment with abstract concepts. Along these lines, Barca et al. (e.g., [160,161]) designed two different studies in which they tested the relation between the extensive use of an oral device (i.e., the pacifier) and abstract conceptual knowledge in children. While some studies (e.g., [162,163]) have found evidence for an impairment in emotional competence (e.g., expression and recognition of emotions in faces) as a consequence of an extensive use of pacifiers, the link between the latter and abstract concepts has still been unexplored. In a first study by [160], children aged 6–7 years with different histories of pacifier use (ranging from never to three years of use) were asked to produce oral definitions for abstract, emotional, and concrete concepts. Then, the definitions were coded both for accuracy and for the conceptual relations they were composed of. We found no differences in accuracy, but reported some interesting qualitative differences among children depending on their use of the pacifier. Specifically, children who overused the pacifier (i.e., for more than three years) overall, tended to use more examples and functional associations and fewer experiential and free associations to describe concepts than the other children. More importantly, their definitions of abstract and emotional concepts were less sharply diversified from their definitions of concrete concepts as compared with the other groups. This pattern was further confirmed by a second study by [161], in which children (7–8 years old) with differing histories of pacifier use completed a semantic categorization task that included in the target stimuli abstract, emotional, and concrete concepts. The results showed that children who made extensive use of the pacifier during infancy were also slower in the categorization task, and this held especially with abstract concepts. Overall, these results suggest that limiting the mobility of speech motor acts by forcing mouth muscles into a static position for a long period of time during language acquisition might interfere with the subsequent ability to master abstract concepts.

### 3.4. Abstract Concepts and Inner Speech

Whether the mouth sensorimotor system is interfering or facilitating the processing of abstract concepts and words, its involvement is undoubtedly marked. In the context of searching unifying principles to explain different phenomena, this bears the question of which kind of mechanism might underlie the mouth motor activation during the processing of abstract concepts. One reliable hypothesis proposes that the mouth motor activation might be related to inner speech (IS), which could represent the neuropsychological function contributing to the processing of complex and abstract meaning. Over time, IS has been defined differently (for a review, see [164]). Some have defined IS as an initial outer speech, internalized during cognitive development [165]; others described it as an active rehearsal mechanism, using offline speech to plan overt speech or action [166]. Recently, it has been proposed that IS might represent a simulation of articulatory actions recreating auditory percepts fulfilling a self-regulatory behavioral goal ([167], for reviews, see e.g., [157,168,169]). In keeping with the “embodied simulation” theories (e.g., [170]), IS and overt speech seem to overlap partially, and IS might be considered to be the internal preparation for specific (linguistic) motor acts [164]. Behavioral evidence supports such a view. It has been shown that silent reading entails the covert articulation of the speech gesture arranged to produce a particular sound [171,172]. Overall, the literature converges in showing the critical role inner speech might play in improving cognitive processes. For example, recent computational work with a model reproducing the effects of the Wisconsin Card Sorting test showed that inner speech strongly enhanced cognitive flexibility [173]. Consistently, the reduced use of IS could explain some impairments in children and elderly autistic people [174].

In keeping with the Words as Social Tools (WAT) theory (e.g., [11,84,144]), abstract concept acquisition takes place primarily within social contexts through linguistic exchanges. One of the pillars of this theoretical proposal is the social metacognition hypothesis (e.g., [11,144]) that the more abstract and complex concepts are, the more we develop the metacognitive awareness of the limits of our knowledge [175]. Therefore, we would need to use inner speech to retrieve and re-explain to ourselves the meaning of words or prepare ourselves to ask other social actors [11]. Because of the higher uncertainty of word meaning, IS might be more likely to be used in the semantic search of abstract words as compared with concrete words, helping us to collect scattered information to determine what the word really means (e.g., [176,177,178]). Thus, IS might represent the gateway to access complex semantic meanings, which cannot be fully experienced through the five senses.

Here, a possible objection might arise. While reading the newspaper or talking to someone, do we really need to talk to ourselves to search for abstract words? It may seem that comprehension occurs too rapidly and efficiently for this to occur, except in rare circumstances. While further research is needed to address this point, there is evidence showing that inner speech might be condensed (review in [157]), and that inner articulation might be much faster than outer articulation. For example, Korba [179] used self-reporting to assess the rate of inner speech during mental solving of verbal problems (elliptic inner speech). Then, the reported rate was compared with physiological (electromyographical) measures of subvocal activity during problem solving (extended inner speech). Extended word counts were much faster, exceeding the elliptic word counts by 4000 words per minute. While this number might seem impressive, it is possible that impoverished words or initials that in outer speech might seem useless, carry much information for the person using inner speech.

Many studies have attested (review in [157]) that inner speech requires articulation. If abstract concepts, more than concrete concepts, require inner speech for their processing, and inner speech has an articulatory component, then, interfering with the latter might be more detrimental for abstract than concrete concepts. We addressed this question in a recent study [176] where we disrupted the phonological loop, formed by inner speech and the articulatory system (see [180]), in a semantic categorization task with abstract and concrete words. We took advantage of articulatory suppression, i.e., number, word, or syllable repetition, which has been widely used to interfere with the inner speech in cognitive tasks [181,182]. Participants were asked to evaluate whether the words were abstract or concrete by pressing two different pedals with their foot. The experiment included three conditions: a baseline, an articulatory suppression condition, and a manipulation condition. In the last two conditions, participants had to continuously repeat a syllable (articulatory suppression) and manipulate a softball with their dominant hand. The results indicated that the articulatory suppression significantly impacted the processing of abstract words as compared with concrete words, while in the manipulation condition, the magnitude of the effect was reduced. Such evidence supports the idea that abstract concept processing might rely more on linguistic components than on concrete concept processing. It also suggests that social metacognition might be mediated by inner speech [11]; we presumably talk to ourselves through internal dialogue to better master complex meanings or to ask someone for a linguistic contribution to dispel uncertainties. However, processes entailing inner speech do not exhaust the complexity of grounded mechanisms accounting for abstract concept representation. In the following sections, we provide evidence detailing how more situated, however, as we will claim, primarily embodied processes contribute to the mastering and refining of our abstract conceptual repertoire.

### 3.5. Grounding Abstract Concepts in Social Interactions

While concrete concepts can be easily understood by experiencing their referents through the body, the grounding in the sensorimotor system apparently fails to compellingly explain abstract concepts without the integration of other systems such as the linguistic system. Abstract concepts are acquired and mastered through language, a sophisticated skill, or as it has been defined, a “self-constructed cognitive niche” [183] grounded in sociality. Transmitting a meaning implies selecting relevant features of objects to form a labeled, and thereby recognizable category. When creating a category related to a concrete entity, i.e., “cat”, we usually extract common features from perceptually similar exemplars located under the same semantic umbrella. We distinguish this process (i.e., abstraction) from the process that leads to the formation of abstract concepts (i.e., abstractness) [144]. The members of categories such as “justice” or “democracy” do not have many common features and are fairly heterogeneous (low dimensional categories, [142]), and typically, we cannot rely solely on our perceptual system to detect their similarities. Since abstract concepts are among the most complex expressions of the interconnection between language and thought, they are challenging for philosophers, psychologists, and linguists. While, in the case of concrete concepts, perceptually experiencing objects is a crucial step for creating and updating our basic knowledge of the world; negotiation and social exchanges seem to be the dynamic substratum of abstract concepts (e.g., [11,84,184]). As already mentioned, according to the WAT proposal, the activation of mouth motor areas in abstract concept representation might be related to the social origin of abstract concepts. Specifically, we suggest that one of the mechanisms potentially underlying this documented pattern may be the preparation to complement our knowledge by asking someone reliable to provide an explanation, or to validate a meaning (social metacognition (e.g., [11,144,175]). Such social validation of abstract concepts can be either vertical or asymmetric, such as in the case of a child who asks the teacher the meaning of a word, or horizontal or symmetric, as in the case of two peers discussing a concept. This two-folded notion of social metacognition in which the contribution of others is not only intended in terms of hierarchically ordered linguistic exchanges, but also as the symmetrical negotiation and co-construction of meanings, helps us to unravel a further possible mechanism leading to the relevance of language and sociality in abstract conceptual knowledge. In fact, in both cases, individuals must be successfully coordinated to share and verbalize a meaning, indeed, language and communication can be considered joint actions [185]. This aspect is especially evident in discussions that take place among peers concerning, for instance, politicized concepts. To illustrate, consider a concept such as freedom, which is an abstract concept, its definition encompasses several different situations; and therefore it can be flexibly renegotiated and articulated for contextual purposes [186]. Currently, for instance, it is debated whether enforcing laws promoting vaccination against COVID-19 represents a limitation to basic principles of personal freedom. Therefore, the concept of freedom is constantly updated and revised in light of social changes, and this process of redefinition is made possible by social actors discussing and negotiating its meaning. Importantly, discussions available to the general public through social media, and social exchanges such as casual conversations amongst peers, might all contribute to the grounding and reinforcement of specific abstract concepts. These mechanisms can both be condensed into the idea of social metacognition as a grounding source. In fact, we hypothesize that when we retrieve the concept of freedom, linguistic, and social experiences related to both vertical and horizontal social validation of meanings are re-enacted to refine our conceptual repertoire. In two recent studies carried out in our lab with children and adults, we found evidence outlining the crucial role of sociality in the grounding of abstract concepts. In the first study [187], we employed thermal imaging, while 5–7-year-old children were asked to decide whether a series of prerecorded words were Italian or not, and to respond by pressing a button on the laptop keyboard (lexical decision task). If they did not know the meaning of the words, they had to refrain from responding. The response time analysis revealed that children employed more time to process abstract words as compared with concrete words, indicating a concreteness effect in response times also in very young children. More crucially, thermal imaging results revealed that the parasympathetic system of children was more active when they were presented with abstract words than it was when children attended to concrete words. Importantly, this system has been associated with prosocial behaviors [188]. Such evidence fosters the idea that we acquire abstract concepts in a social context, and that the presence of others is crucial to validate complex meanings.

In the second study implemented through kinematics techniques [189], participants were asked to predict the actions of an avatar on the screen in order to plan their own actions towards a bottle-shaped object (joint action task) (e.g., [190,191]). The participants could freely choose how to grasp the object (they could use either the upper part with a precision grip or the lower part with a power grip), but they were asked to perform either imitative or complementary actions with respect to the avatar’s actions. Therefore, for example if the avatar used a precision grip, they could either grasp the object using a precision grip, or they could use a power grip. Before and after the joint action task, participants took part in a concept guessing task, which consisted of guessing the concept evoked by a visual image displayed on a screen. Two confederates provided participants with hints to guess the correct concept associated with the image. One confederate helped participants to guess abstract concepts, and a second confederate helped them to guess concrete concepts. Crucially, the experimenter manipulated the participants’ beliefs about the avatar’s identity, and therefore they believed that interacting with an avatar embodying the confederate was helping them to guess abstract or concrete concepts. The results showed that participants asked for more hints for abstract concepts as compared with concrete concepts. In addition, participants were also aware of their higher need for help from others when guessing the meaning of abstract concepts as compared with concrete concepts. Therefore, the metacognitive feeling or assessment of the limits of their knowledge might have led participants to rely more on available social actors and to show more deference [175]. Moreover, data from the human–avatar motor interaction task showed that the need to rely on others influenced participants’ abilities to interact. Participants’ performances were more synchronous with the avatar embodying the confederate associated with guessing abstract concepts than with the confederate associated with concrete concepts. This last result suggests that during verbal interactions involving abstract concepts, linguistic actors are particularly tuned in for building up new insights on complex meanings. Remarkably, this fine attunement entails a physical and bodily synergy that might support and enhance linguistic exchanges.

Theories of cognitive evolution suggest that the development of abstract representations might respond to the need of being connected with conspecifics (e.g., [192,193]). Sharing abstract concepts entails a deep agreement of thoughts among individuals adhering to a system of values within a community. Indeed, social cohesion within and among groups is created and maintained through a dynamic network of co-built knowledge. In this sense, abstract concepts are not only the “glue” holding together scattered and heterogeneous information, but they also represent a “social glue” providing a common reference of knowledge within societies [194]. If abstract concepts are intrinsically social in their origin and function, we can expect that any verbal interaction with abstract content might promote a sense of “psychological closeness”, provided that social actors draw from the same source of collective knowledge. Recently, we tested this hypothesis in a study [195] where participants were asked to write sentences through an online platform starting from abstract and concrete concepts. In one condition (i.e., “social condition”), participants conversed in dyads. In another condition (i.e., “individual condition”), they wrote sentences cued by abstract and concrete words independently, but knowing that on the other side of the screen, another person was doing the same, and that later they would read what the other had written. After each conversation or verbal production, we measured psychological distance using the Inclusion of Other in the Self (IOS) scale [196]. We found that conversing increased the psychological closeness between participants as compared with the condition in which participants were not conversing, regardless of the abstract or concrete content of the verbal production. Looking at the results of the “social condition” alone, we found that conversations on abstract concepts were perceived as more demanding as compared with conversation on concrete concepts, and that the contribution of the paired participants was deemed to be more relevant in the case of abstract conversations. These findings suggest that in conversations prompted by abstract concepts, the contribution of others might be perceived to be necessary because of the spontaneous dialogical approach to master abstract, complex meanings. Moreover, the results indicated that the higher the other’s contribution in the conversation about abstract concepts was rated, the more the perceived psychological closeness increased between the interlocutors, while this was not the case for conversations elicited by concrete concepts. These results seem to be in line with the social metacognition proposal, according to which when mastering complex and abstract meaning, we might prepare ourselves to a constructive dialogue to dispel ambiguities and increase the mental connection with the interlocutor. Finally, the role of linguistic and social interaction for abstract concept processing has been corroborated by a study in preparation [197]. Participants categorized different kinds of concrete (tools and food) and abstract words (theoretical and institutional concepts) primed by images representing social-action (dancing together), linguistic-social (dialogue), and linguistic-textual (reading a book) situations, and a control condition (landscape). As predicted, the critical primes, but not the control one, modulated the processing of abstract but not of concrete concepts, slowing down response times. We interpreted the results arguing that the linguistic and social experiences activated by the prime might conflict with similar resources necessary to form a simulation of a word’s meaning. Overall, the evidence coming from these recent studies suggests that social interactions may be a determining grounding source promoting abstract concept acquisition and evolution.

## 4. Conclusions

In this paper, we address the role played by the sensorimotor system across various processes, including perception and recognition of objects, conceptual acquisition, and abstract concept and word processing. The involvement of the sensorimotor system at these different levels clearly indicates that the traditional distinction between low-level processes such as perception, and high-level processes such as conceptualization and language, does not hold. Our results fit perfectly with theories of reuse [2,3], according to which higher-level systems, such as language, build on lower-level systems. Once demonstrated and taking for granted the pivotal role of the sensorimotor system, the main suggestion of our contribution is that future research should better understand how and to what extent this system is involved in different processes. Here, we focused on two aspects that we believed might offer precious insights into this pursuit and have illustrated their importance in light of recent evidence in our lab and in labs with which we collaborated. The first aspect is the flexibility of the engagement of the sensorimotor system under multifarious circumstances. We have seen through examples on affordances that their activation, and hence the involvement of the sensorimotor system, was strongly influenced by physical and social context. The second aspect is the different levels of involvement of the sensorimotor system, the role of which can be integrated and flanked by that of other systems. As we fleshed out throughout the paper, when abstractness increases, concepts are more detached from sensory modalities, and language acquires a prominent role. However, this does not rule out sensorimotor components. On the contrary, we discussed studies providing evidence that varying sensory modalities are still active, even if to a lesser extent or in different forms. Understanding the mechanisms underlying this flexibility and the level of involvement of our sensorimotor system represents, in our view, two significant challenges for future research.
